# Associations among Different Internet Access Time, Gender and Cyberbullying Behaviors in Taiwan’s Adolescents

**DOI:** 10.3389/fpsyg.2017.01104

**Published:** 2017-06-30

**Authors:** Cheng-Min Chao, Tai-Kuei Yu

**Affiliations:** ^1^Department of Business Administration, National Taichung University of Science and TechnologyTaichung, Taiwan; ^2^Department of Business Administration, National Quemoy UniversityKinmen, Taiwan

**Keywords:** adolescent, cyberbullying behavior, attitude toward cyberbullying, internet use, partial least squares (PLS)

## Abstract

With the increasing convenience of social networking sites and their interconnectedness with human interaction, verbal and image bullying have turned digital, making cyberbullying a new form of bullying attracting the attention of researchers, social workers, and schools. This study focuses on the status quo of attitude toward cyberbullying and cyberbullying behavior, explores associations of attitude toward behavior on cyberbullying behavior in gender and different internet access times. In a cross-sectional survey, adolescents were surveyed through self-report questionnaires, 13,864 respondents were received among 150 high schools in Taiwan. Statistical analyses were performed using structural equation modeling (SEM). The study revealed that attitude toward cyberbullying has a direct effect on cyberbullying behavior; among the greatest direct impact were when students use the Internet during the time frame 10:00–14:00. Parents and schoolteachers pay special attention to students using the Internet during this time frame 10:00–14:00 and give guidance, express care, and help those being bullied to communicate and use the Internet in a correct manner. Among genders, male student attitude toward behavior has a greater effect than those of female students. Both male and female students know what cyberbullying is and have witnessed, heard of, or personally encountered cyberbullying behavior. We recommend students talk to parents or teachers or other people who care to reduce the negative effects of cyberbullying. We hopeful that the conceptualization model presented in this study serves as an activator for researches on attitude toward cyberbullying and cyberbullying behavior, and serves a guide and a call to attract more researches in this area.

## Introduction

With the advancement of technology and the widespread access to the Internet, the Internet has revolutionized the way in which people connect and communicate lives (Chao et al., 2013; Pawlikowski et al., 2014; Good and Fang, 2015; Quinones and Kakabadse, 2015; Wegmann et al., 2015; Hsieh et al., 2016). According to the Taiwan Communication Survey (2015) Internet project reported findings from a national survey of about ninety percent of Taiwanese young people between 9 and 17 years are on the internet and this statistic has been on the rise (Taiwan Communication Survey, 2015). These young people spend about an average of 3 h per day on the Internet. And 14 year old teenagers spend the longest time about an average of 3.5h online per day (Taiwan Communication Survey, 2015). In the past decade, the exponential growth of information technology and the widespread access to the Internet, adolescents have tended to spend more time in the cyber world. Internet provides new information, social-related information, and social networking opportunities but also simultaneously contains risks and a serious form of misbehavior among adolescents, such as: Internet addiction, cyberbullying, cyber pornography, health risks, Internet fraud and cyber victimization that can hurt and distort an adolescents’ development (Bauman et al., 2013).

Cyberbullying and Internet addiction are a serious issue for young people in the World. The prevalence rates of cyberbullying vary based on the population studied (i.e., gender, age group), the form of cyberbullying is being measured, and the measure is used to assess cyberbullying incidents. Of note, the majority of cyberbullying studies have focused on youth between the age of 10 and 17 (Kowalski and Limber, 2007; Yılmaz, 2011; Chang et al., 2013; Cleemput et al., 2014; Özdemir, 2014; Taiwan Communication Survey, 2015). Cyberbullying among adolescents has emerged as a new form of bullying and has attracted the attention of many researchers in recent years because the ratio of adolescents using the Internet has increased quickly, especially in social networking sites, chat rooms, and instant messaging applications. Adolescents feel a sense of solidarity and identity through the aforementioned applications (Anderson et al., 2014; Dredge et al., 2014; Palladino et al., 2015). Cyberbullying behavior and traditional school bullying have some commonality (Bauman et al., 2013; Waasdorp and Bradshaw, 2015). In fact, it has been found that cyberbullying may lead to more severe negative outcomes when compared to traditional bullying. From a traditional point of view, the perpetrators of cyberbullying use online videos, pictures and words in digital form to threaten, ridicule, and insult others. Research suggests that cyberbullying may negatively impact multiple aspects of adolescents mental health includes: depression, social anxiety, suicide, low self-esteem, etc; and behavioral problems include deterioration of relationships between family members and a drop in grades and other negative outcomes (Anderson et al., 2014; Dredge et al., 2014). However, not all victims of cyberbullying have had a negative repercussions (Patchin and Hinduja, 2006; Burgess-Proctor et al., 2009); exploring the consequences of cyberbullying is also rather important.

Cyberbullying is defined as the behavior of using modern communication technology to post pictures, videos, communication device [including: mobile phones, social networking sites (SNS), instant messenger (IM), e-mail, facebook, blog, etc], or texts that ridicule, insult, threaten, or intimidate a person and cause feelings of hurt in said person (Huang and Chou, 2010, 2013; Anderson et al., 2014; Cleemput et al., 2014; Hong et al., 2014; Watts et al., 2017). In a United States sample of 3,767 middle school students in 6th-8th grades (aged 11–14), around 11% of 6th-8th grade students had cyberbullied others within the 2 months prior to completing the survey; 4% had cyberbullied someone (cyberbully); and 7% had cyberbullied others and had also been both cyberbullies and victims (Kowalski and Limber, 2007). The Taiwan cyberbullying of adolescents project reported findings from a national survey of 1,959 students (ages 9–17). Of the respondents, 5.8% said that they had been the victims of cyberbullying, whereas 8.3% said that they had engaged in bullying behavior online (Taiwan Communication Survey, 2015). Among all the ways of youth cyberbullying, social media (e.g., Facebook or Plurk) is the most common one. About 2/3 (68.7%) of the interviewees said that they had been bullied on social media, and about 1/2 (42.3%) had been bullied when playing online games. Even on IMs, chatrooms, forums, BBS (Bulletin Board System), cyberbullying is a frequently occurred incident (Taiwan Communication Survey, 2015). There is approximately 1 among 4 people who had been bullied while using the above mentioned online services (IMs: 25.6%; chatrooms, etc.: 24.8%) (Taiwan Communication Survey, 2015). Ditch the Label (2013) released its annual cyberbullying report which combines the largest bullying-related data set of over 10,000 young people with key questions surrounding cyberbullying and the use of integrated digital technology within the lives of young people. In cyberbullying and social networks issue, of all youths polled, 75% of them use facebook and 54% have experienced cyberbullying. In addition, this report also revealed that cyberbullying happens mostly on Facebook, Youtube, Twitter, and Myspace. Ditch the Label (2016) surveyed 8,850 young people aged 12–20 in partnership with schools and colleges from across the all across the UK. The results showed that people who have been bullied are almost twice as likely to bully others; boys bully twice as more as (66% of males versus 31% females); 44% of young people who have been bullied experience depression; 33% of those being bullied have suicidal thoughts. The ease of producing information online, fast and widespread dissemination, coupled with anonymous posting, and the elimination of the pressures of face-to-face confrontation have made it easier and quicker for a cyberbully to hurt others. The evolution of this new form of bullying deserves the attention of educators because this kind of bullying and oppressive behavior causes great harm to students and adolescents undergoing personality development.

Past studies have shown that the effects of cyberbullying are more severe than traditional bullying in four different ways: (Anderson et al., 2014; Dredge et al., 2014) (1) the identity of the perpetrator of cyberbullying is unknown; (2) the number of bullies is greater; (3) the rate of information sharing is faster; (4) the record of bullying lasts forever, much like insulting a person repeatedly. With cyberbullying, there is no direct way for perpetrators to know the effect of their behavior on the victim. Hence, the victim may feel terror, pressure, and other adverse effects when the victim does not know the identity of the perpetrator. When the victim is under pressure, there is a higher probability for violent behavior (Huang and Chou, 2013; Anderson et al., 2014; Hong et al., 2014).

Existing empirical research have examined the influence of cyberbullying and victimization among adolescents. The results show that parenting behavior, good family relations (such as: parental monitoring, positive parent–adolescent relationships and communication) can decrease involvement in risky activities of cyberbullying (Law et al., 2010; Stadler et al., 2010; Özdemir, 2014). But, adolescents tend not to share negative experiences with their parents. For instance, Tokunaga (2010) found that although about 20–40% of adolescents are victims of cyberbullying, most choose not to tell their parents. Among the victims, suicidal tendency is higher than among non-victims. Also, 16% of adolescents admitted to cyberbullying behavior once or regularly. Yılmaz (2011) found that about 62% of students who had been cyber bullied did not discussed the problem with parents or teachers. In a recent study of 2,992 Taiwan high school students in 10th grade, 18.4% had been cyberbullied (cybervictim); 5.8% had cyberbullied others (cyberbully); 11.2% had both cyberbullied others and been cyberbullied (cyberbully victim) (Chang et al., 2013). According to findings published from the Taiwan Communication Survey (2015), middle and high school students are more likely to become victims of cyberbullying. Also, among 9–17 year olds in Taiwan, 5.8% have been victims of cyberbullying and 8.3% have bullied others. The most common channels were social media sites and online games. When faced with cyberbullying, 36.7% of the adolescents surveyed ignored the behavior. For example, during information class, one high school student, May, learned about attitudes toward cyberbullying and grasped a deeper understanding as the teacher frequently applies real world cases to teach students the definition of cyberbullying and how to deal with such behavior in her information course. Sometimes May witnesses classmates bad-mouthing another classmate or sending photos or videos mocking said classmate. May has realized that cyberbullying does exist in real life from her observation of online news stories on the negative effects of cyberbullying on artists and students. As May increasingly used the Internet, she saw more cases of cyberbullying and understood the contexts of cyberbullying.

Behavioral beliefs can vary from individuals’ attitude toward the behavior. In other words, individuals with a more positive attitude toward a certain behavior tend to perform the behavior. Ho et al. (2017) explored factors related to cyberbullying on social media among children and adolescents in Singapore. The results showed that individuals with less favorable attitude had a lower likelihood to engage in cyberbullying behavior. Doane et al. (2014) explored cyberbullying perpetration among American college students. The results presented strong positive correlations between attitude, subjective norms, and cyberbullying perpetration. Many previous studies have found that attitude toward cyberbullying is significantly associated with cyberbullying (Perren and Gutzwiller-Helfenfinger, 2012; Doane et al., 2014; Ho et al., 2017).

About the relationship between cyberbullying and genders of adolescents. Previous research investigating the significant differences of the incidences of cyberbullying displays inconclusive findings. Huang and Chou (2010) argue that gender has long been associated with aggressive behaviors, and it may result in different types of bullying among adolescents. Some researchers found no significant differences of cyberbullying experiences between male and female youth (e.g., Ybarra and Mitchell, 2004; Similarly, Patchin and Hinduja, 2006), while others do not support this view (e.g., Kowalski and Limber, 2007). Some researchers have shown that males are more likely to be perpetrators of cyberbullying than are females (Huang and Chou, 2010; Chang et al., 2015; Lee and Shin, 2017). In conclusion, the relationship between adolescent cyberbullying and the genders seem ambiguous. Therefore, further investigation is necessary. In addition, most prior research of cyberbullying has focused on the factors and the influences it has on mental and physical health (Chang et al., 2013; Anderson et al., 2014; Dredge et al., 2014; Hong et al., 2014) and the differences between cyber and traditional bullying. With the Internet become more common, and people spend more time online, cyberbullying, especially between students, has become an important topic. However, there is a lack of examinations how different attitudes toward cyberbullying of students with different levels of Internet addiction affect their cyberbullying behaviors.

In recent years, adolescent Internet addiction has emerged as a major issue. Internet addiction is the inability of people to control their Internet use—an inability that can eventually lead to be a global major mental health, social, and academic problems (Wegmann et al., 2015; Chen and Nath, 2016; Hsieh et al., 2016; Montag et al., 2016; Musetti et al., 2016; Servidio, 2017). The prevalence of youth Internet addiction varies widely across countries, with the early studies of Internet addiction reported findings from a survey conducted in 11 European countries, taken by about 11,000 adolescents (mean age: 14.9 ± 0.89). The results showed that the overall prevalence of Internet addiction was 4.4%, higher among males than females (5.2% versus 3.8%) (Durkee et al., 2012). In Poland, about 2–9% of young people have very serious problems caused by Internet usage (Hawi et al., 2015), and the prevalence in Hong Kong at between 6.7 and 26.7% (Wang et al., 2015). According to the survey of Taiwan Network Information Center [TWNIC] (2015), during the last 6 months, 79.7% of Taiwanese people aged 12 and more who use the Internet have spent approximately 3.42 h online per daily, and 3.35 h per daily during holidays. The most common Internet activities are visiting online communities and using IMs (Taiwan Network Information Center [TWNIC], 2015). Internet addiction prevalence estimation varies across countries due to differences in diagnostic criteria as well as the psychometric tools utilized for assessment (Hawi et al., 2015; Boysan et al., 2017). Kuss et al. (2014) found that there are currently 21 existing instruments for measuring Internet addiction. Current screening scales of Internet Addiction are mainly adopted from Young’s Internet addiction Scales (Young, 1998), which appeared as one of the earliest conceptualizations and is still being commonly adopted nowadays. Taiwan’s popular Chinese versions of Internet addiction scales also uses the symptoms of DSM-IV-TR Diagnostic Criteria, for substance addiction as the diagnostic indicator (Ko et al., 2005; Yen et al., 2009). To conclude, Internet addiction among teenagers is a relatively serious problem. A comprehensive Internet addiction measuring tool might effectively categorize the addiction levels. In addition, previous research seldom covers Internet addiction together with cyberbullying. It is especially important to examine the influence of different Internet addiction levels on the cyberbullying behaviors.

To summarize, cyberbullying gives birth to a new kind of public physical and mental health problem that negatively impacts the everyday life of a substantial number of adolescents. The effect of attitude toward cyberbullying on cyberbullying behavior is an important topic for discussion. Therefore, the purposes of the present research are (1) to investigate antecedent (attitude toward cyberbullying) for cyberbullying behaviors, and (2) to examine the effects of gender, most frequent Internet access time, and Internet addiction situations on cyberbullying behaviors. In order to understand the association between attitudes toward cyberbullying and cyberbullying behavior modulates by gender and different internet access. This study aims to address the following research questions: (1) Does attitudes toward cyberbullying influence the cyberbullying behaviors? (2) How does the gender, most frequent Internet access time, and Internet addiction the impacts of cyberbullying behaviors? To minimize cyberbullying, the results of this study provide references for parents, schools, and government educational units, further adding emphasis on the effects of cyberbullying and the problems raised.

## Materials and Methods

### Hypotheses and Research Model Development

In order to understand the association between attitudes toward cyberbullying and cyberbullying behavior modulates by gender and different internet access. The purposes of the present research are (1) to investigate antecedent (attitude toward cyberbullying) for cyberbullying behaviors, and (2) to examine the effects of gender, most frequent Internet access time, and Internet addiction situations on cyberbullying behaviors.

According to the previous research (Perren and Gutzwiller-Helfenfinger, 2012; Doane et al., 2014; Ho et al., 2017) pointed out that positive correlations between attitude toward cyberbullying and cyberbullying behaviors. Consequently, this appears to support that attitude toward cyberbullying is the strongest predictor of cyberbullying behaviors. In addition, the effect of gender on cyberbullying is controversial. Some researchers have shown that males are more likely to be perpetrators of cyberbullying than are females. Other researchers, however, indicate no gender effect on cyberbullying behaviors (Ybarra and Mitchell, 2004; Similarly, Patchin and Hinduja, 2006; Roberto et al., 2014).

On a global scale, the reported prevalence estimates for adolescent Internet addiction vary between 2% (Poland) and 26.7% (Hong Kong) (Hawi et al., 2015; Wang et al., 2015). Thus, adolescent Internet addiction has emerged as a major issue. Although there exists extensive research in the field of Internet addiction, most researches are lacking in cyberbullying. It is especially important to examine the influence of different Internet addiction levels on the Internet bullying behaviors. Based on the abovementioned considerations, we advance the following hypotheses:

Hypotheses 1: Attitude toward cyberbullying has a significant effect on cyberbullying behaviors.

Hypotheses 2: The moderating effect of gender exists between the Attitude toward cyberbullying and cyberbullying behaviors.

Hypotheses 3: The moderating effect of most frequent Internet access time exists between the Attitude toward cyberbullying and cyberbullying behaviors.

Hypotheses 4: The moderating effect of Internet addiction exists between the Attitude toward cyberbullying and cyberbullying behaviors.

### Measures

This study used questionnaires as the main instruments for data collection. In the questionnaires, the instrument was designed 38 questions with a four-part questionnaire, includes: The content of the questionnaire includes four components: (1) basic information of the student and Internet usage; (2) attitude toward cyberbullying (1 = strongly disagree, 4 = strongly agree), (3) cyberbullying behavior (1 = not at all, 4 = very often), and (4) Internet addiction (1 = strongly disagree, 4 = strongly agree). The Likert four point scale was used in the “Attitude toward Cyberbullying,” “Cyberbullying Behavior,” and Internet addiction sections. The self-reporting inventory was used for filling out the questionnaire. The questionnaire used is shown in **Table 1**. Due to the nature of the questions in “Cyberbullying Behavior,” there is a tendency for participants to avoid them, causing common method variance (CMV). We chose to write the questions from a third party perspective to evaluate stories the respondents have heard or saw about cyberbullying. We undertook a large sample to avoid CMV and to increase the reliability power of this study.

**Table 1:** Questionnaire items.

Construct	Items	Descriptions
Attitude toward cyberbullying	ATC1	If I saw hateful content against others online, I would tell my teacher.
	ATC2	If my classmate bullied others online, I would not intervene in order to protect myself.
	ATC3	If I were bullied, it is safer to express my resentment online.
	ATC4	If I were attacked/mocked online, it is fine to fight back anonymously.
Cyberbullying behaviors	CB1	My classmate once made rude comments about other classmate online.
	CB2	My classmate once made rude comments about me online.
	CB3	My classmate once posted derisive images or media online.
	CB4	My classmate once held online polls to humiliate others.
	CB5	I have seen videos online of someone being bullied.
	CB6	I have seen people verbally attacking each other online.
Internet addiction	IA1	Do you feel preoccupied with the Internet when off-line, or fantasize about being online?
	IA2	Do you form new relationships with fellow online users?
	IA3	Do you feel depressed, moody, or nervous when you are off-line, which goes away once you are back online?
	IA4	Do you lose sleep due to late-night log-ins?
	IA5	Do you fear that life without the Internet would be boring, empty, and joyless?
	IA6	Do you feel restless, moody, depressed, or irritable when attempting to cut down or stop Internet use?
	IA7	Do you find yourself saying “Just a few more minutes” when online?
	IA8	Does your work suffer because of the amount of time you spend online?
	IA9	Do you feel the need to use the Internet with increased amounts of time in order to achieve satisfaction?
	IA10	Does your school work or friend relationship suffers because of the amount of time you spend on-line?
	IA11	Do you check your e-mail before something else that you need to do?
	IA12	Do you find that you stay online longer than you intended?
	IA13	Do others in your life complain to you about the amount of time you spend online?
	IA14	Do you choose to spend more time online over going out with others?
	IA15	Do you prefer the excitement of the Internet to intimacy with your friends or family?
	IA16	Do you find yourself anticipating when you will go online again?
	IA17	Does your job/school performance or productivity suffer because of the Internet?
	IA18	Do you try to cut down the amount of time you spend online and fail?
	IA19	Do you block disturbing thoughts about your life with soothing thoughts of the Internet?
	IA20	Do others in your life complain to you about the amount of time you spend online?

The instruments were developed after a thorough review of several previous studies on attitude toward cyberbullying, cyberbullying behavior, and Internet addiction. Our scale development followed MacKenzie et al. (2011) and the development procedures suggested by DeVellis (2003) for standard psychometric scales. The measurement items for the constructs of attitude toward cyberbullying and cyberbullying behavior were adapted from the measurement developed by previous studies (Yılmaz, 2011; Chang et al., 2013; Anderson et al., 2014; Cleemput et al., 2014; Dredge et al., 2014). To define adolescents Internet addiction scale, which was modified from the Chen Internet Addiction Scale (CIAS), Internet Addiction Test (IAT) and other measurement tool about Internet Addiction (Young, 1998; Ko et al., 2005; Kuss et al., 2014; Hawi et al., 2015; Wang et al., 2015; Chen and Nath, 2016; Hsieh et al., 2016; Boysan et al., 2017; Servidio, 2017) to measure Internet addiction.

A pilot test using the questionnaire was conducted on 1,225 senior high school students (including vocational high school students) in central Taiwan to evaluate the revised questionnaire in terms of readability, ease of understanding, and formatting issues prior to the actual test. Further, a Cronbach’s alpha test was performed to test the reliability and internal consistency of each of the 32 measured attributes. The alpha coefficients for all of the 32 attributes ranged from 0.62 to 0.74, which respectively exceeded the minimum value of 0.6 that is widely used as an indicator of reliability (Hair et al., 2010). Subjects who participated in the pilot test were excluded from the subsequent study.

### Participants

In the Taiwanese educational system, elementary, junior and senior high school education is compulsory, children enter the elementary school at the age of 7 or 8 years and complete this stage of their education by the age of 13 or 14 years. Then, they enter junior high school for 3 years followed by a further 3 years at senior high school (including vocational high school). The years at junior high school are termed as the 7th–9th grade, and those at senior high school (including vocational high school) are termed as the 10th–12th grade. To understand the effect of attitude toward cyberbullying on cyberbullying behavior among senior and vocational high school students in Taiwan. As of May 2014, 344 senior high schools and 155 vocational high schools (499 in total) were registered. The sample was first stratified by region (northern, central, southern, and eastern), then stratified by school type (senior high schools and vocational high schools). This cross-sectional survey was conducted in Taiwan among 17- to 19-year-old senior and vocational high school adolescents. Participants were recruited from 150 high schools in Taiwan through stratified and random cluster sampling. Of these, 103 senior high schools (selection rate, 29.9%) and 47 vocational high schools (selection rate, 30.3%) were selected. At every school, 100 students were surveyed through questionnaires.

This research adopted a quantitative survey and utilized mail or face-to-face interviews for high schools that were willing to participate in the survey. The first step, we contacted a teacher who service in academic affairs in each high school to ensure his/her cooperation. They helped explain the content of the questionnaire to the respondents, the duration of data collection took around 8 months. A total of 15,000 questionnaires were sent at the same time. All responses to the self-report instruments were collected during a regular school-day in classrooms and in the presence of the class teacher. All students enrolled in the sampled schools were the participants of this study. Participation in the study was completely voluntary. In total, 13,864 valid responses were received, and the response rate was 92.4%.

### Statistical Analysis

Structural equation modeling (SEM) was used in a comprehensive, combined analysis of both the measurement and structural model. According to Hair et al. (2011), although the covariance-based structural equation modeling (CB-SEM) has dominated since its first appeared in the 1980s, the partial least square SEM (PLS-SEM) has called a great deal of attention in recent years. PLS is component-based and uses a least-squares estimation procedure. For the actual data analyses, we used the SmartPLS 2.0 M3 software developed by Ringle et al. (2005) to test the research model of both the measurement and structural model. The results of the SEM estimation, including standardized path coefficients for each hypothesized path in the model, significance based on one-tailed *t*-tests, and the amount of variance explained (*R^2^*).

## Results

### Demographic Variables

In total, 13,864 (6,747 were male, 7,117 were female) usable responses were obtained. The average age of the participants was 17.33 years (*SD* = 0.94 years). The first time Internet access for the sample population indicated 39.3% were K3 to K4 grades. Most frequent Internet access time for the sample population indicated 73.6% were 18:00∼22:00. Place of Internet usage for the sample population indicated 92.6% were Home. **Table 2** shows the demographic and Internet usage characteristics of the sample.

**Table 2:** Profiles of respondents (*N* = 13864).

Demographics/Level	*N*	Percentage	Demographics/Level	*N*	Percentage
**Gender**			**First time Internet Access**		
Male	6747	48.7	Prior to Elementary School	1260	9.1
Female	7117	51.3	Grade K1–K2	2375	17.1

**Most Frequent Internet Access Time**			K3–K4 Grades	5448	39.3
2:00∼6:00	69	0.5	K5–K6 Grades	3389	24.4
6:00∼10:00	172	1.2	K7–K9 Grades	999	7.2
10:00∼14:00	437	3.2	After K10 Grades	393	2.8
	
14:00∼18:00	792	5.7	**Place of Internet usage**		
18:00∼22:00	10200	73.6	Home	12835	92.6
22:00∼2:00	2194	15.8	School’s computer center	333	2.4
			Internet cafe	347	2.5
			Library	47	0.3
			Other place	302	2.2

**Item**				**Mean**	***SD***

Age				17.33	0.94
During the past 6 months, average monthly amount of money spent on buying things on the Internet (US$)				5.74	32.07
During the past 6 months, number of friends you have made on the Internet				13.92	51.95
During the past 6 months, number of good friends you have made on the Internet				2.73	13.52

### Measurement Model Evaluation

Using linear structural relational estimations and traditional alphas, the assessment of the measurement model includes three indices: reliability coefficients (Cronbach’s α), average variance extracted (AVE), and composite reliability coefficients. The results presented in **Table 3**, all values displayed a higher Cronbach’s α coefficient than the 0.60 benchmark recommended by Hair et al. (2010). The constructs also exhibited a higher composite reliability than the benchmark of 0.6 (Fornell and Larcker, 1981). That all of the convergent validity were exceeded this criteria, except for behavioral of cyberbullying.

**Table 3:** Validity and reliability.

	Cronbachs alpha	Composite reliability	Average variance extracted (AVE)	*R^2^*
Attitude toward cyberbullying	0.891	0.920	0.696	
Cyberbullying behaviors	0.737	0.820	0.432	0.109

### Hypotheses Testing

In the PLS analysis, the *R^2^* values are used as a goodness-of-fit measure. The construct of attitude toward cyberbullying was significant determinants of cyberbullying behavior (β = 0.33, *t* = 37.33, *p* < 0.01). This research model explained 10.9% of the variance in cyberbullying behavior. **Table 4** shows the results of further investigation: firstly, male students have higher coefficient (β = 0.34, *t* = 25.84, *p* < 0.01) and explanatory ability (*R^2^* = 0.116) in their effects of attitude toward cyberbullying on behavior. Secondly, students surf the Internet during 10:00 to 14:00, which leads to higher coefficient (β = 0.40, *t* = 9.66, *p* < 0.01) and explanatory ability (*R^2^* = 0.161). That reveals attitude toward cyberbullying has more significant impact on cyberbullying behavior for students who access the Internet during 10:00 to 14:00. Third, among different Internet addiction situations, most students are Internet addicted, which leads to higher coefficient (β = 0.28, *t* = 15.75, *p* < 0.01) and explanatory ability (*R^2^* = 0.078). The data reveals that when students are addicted to the Internet, the influence on their cyberbullying behaviors are highly significant.

**Table 4:** Result of SEM analysis.

Level	*N*	Attitude toward cyberbullying (Mean ± SD)	Cyberbullying behaviors (Mean ± SD)	β	*t*-value	*R^2^*
**Gender**					
Male	6747	2.26 ± 0.51	2.01 ± 0.56	0.34	25.84^∗∗^	0.116
Female	7117	2.23 ± 0.51	1.99 ± 0.57	0.32	25.24^∗∗^	0.102
**Most Frequent Internet Access Time**					
02:00∼06:00	69	2.25 ± 0.48	2.06 ± 0.54	0.38	2.36^∗∗^	0.143
06:00∼10:00	172	2.27 ± 0.50	2.07 ± 0.60	0.29	3.98^∗∗^	0.082
10:00∼14:00	437	2.24 ± 0.53	1.99 ± 0.56	0.40	9.66^∗∗^	0.161
14:00∼18:00	792	2.25 ± 0.53	1.99 ± 0.58	0.34	8.94^∗∗^	0.115
18:00∼22:00	10200	2.24 ± 0.51	2.00 ± 0.57	0.33	33.66^∗∗^	0.110
22:00∼02:00	2194	2.25 ± 0.52	2.00 ± 0.55	0.32	14.76^∗∗^	0.099
**Internet Addiction Situations**					
Normal	6668	1.84 ± 0.31	1.85 ± 0.51	0.13	11.45^∗∗^	0.016
Slightly addicted	2773	2.33 ± 0.07	1.98 ± 0.50	0.14	7.62^∗∗^	0.020
Addicted	4423	2.81 ± 0.32	2.22 ± 0.60	0.28	15.75^∗∗^	0.078

*^∗^p < 0.05; ^∗∗^p < 0.01.*

## Discussion

The analysis of this study shows that attitude toward cyberbullying has an effect on cyberbullying. The explanatory power is 10.9%, the reason being when faced with cyberbullying, most are afraid to publicize it, nor do they tell their parents or ask for help. Also, students are unsure of the definition of cyberbullying and are unable to detect cyberbullying when confronted. Some students mistake cyberbullying as making fun of peers, so they do not pay attention, and suddenly realizing the severity of the situation after the effects of cyberbullying have already snowballed. For example, high school student Jason picked up his classmate, Jackson’s, phone. Jason posted indecent photos of Jackson from his phone on the Internet as a joke, unaware that this was cyberbullying behavior. Jackson committed suicide as a result. We recommend teachers remind students to not like, respond, or share posts criticizing or bullying others and to not participate in suspicious search engine activities to avoid being an accomplice to cyberbullying. When a student is faced with cyberbullying or discovering a classmate being cyberbullied, teachers and parents should be notified immediately to put an end to this behavior.

The purpose of this study was to explore the factors associated with cyberbullying behaviors based on attitude toward cyberbullying. In this study, the individuals with less favorable attitude had a lower likelihood to engage in cyberbullying behavior. This is consistent with previous research, where a positive correlation was found between positive attitude toward cyberbullying and cyberbullying behaviors (Perren and Gutzwiller-Helfenfinger, 2012; Doane et al., 2014; Ho et al., 2017). Results also indicated that attitude toward cyberbullying was the key predictor of cyberbullying behavior. Besides, some previous studies (Selkie et al., 2015; Watts et al., 2017) have shown that when one has been a cyberbully or a victim of it, they tend to fall into these same categories in college. Therefore, high school teachers should try to prevent students from being cyberbullies or cybervictims. In order to achieve that, students’ attitude toward cyberbullying is important as it is to the behaviors. Past scholars (Washington, 2014; Watts et al., 2017) suggested instructors must take a more active role in monitoring online interactions to prevent cyberbullying among students.

It is difficult for parents or teachers to monitor the Internet; everyone browsing SNSs or other websites is a spectator, and the number of spectators can multiply, augmenting cyberbullying. Therefore, to deter the growth of online violence, future cyberbullying prevention and relevant education should start with spectators taking action and the establishment of the concept of using the Internet for positive uses. From the school’s perspective, teachers need to understand the motives for cyberbullying. Every online user, regardless of their identity or whether they are an adult or a high school student, should be educated.

Among genders, male student attitude toward behavior has a greater effect than those of female students. Both male and female students know what cyberbullying is and have witnessed, heard of, or personally encountered cyberbullying behavior. The results of the survey show that regardless of gender, when confronted with cyberbullying, students choose to keep to themselves and not ask for help. We recommend victims of cyberbullying to not keep quiet about an incident and to mitigate the negative effects through the care of concerning adults. Cyberbullying is not a short-term phenomenon, but a vicious cycle. One feels the need to retort when being bullied, then the cyberbully strikes back, creating a cycle of bullying. Parents and teachers can inform the students of the correct attitude to deal with cyberbullying and even ask for the school’s help, minimizing the damage done by cyberbullying.

In terms of most frequent Internet access time, students online during the 10:00–14:00 were most affected by attitude toward cyberbullying on cyberbullying behavior. Students utilize this time frame to check the latest status on SNSs as it is their rest time at school. Cyberbullying messages may be intermixed with replies on any given video and photo post. This group is not only at high risk for cyberbullying, they are also considerably addicted to the Internet and unable to stay off the Internet for long periods of time. Because this is during school hours, schools have a stronger capability in disciplining this group. We recommend schools and teachers to pay special attention to and try to understand the browsing habits and online interactions of this group to reduce the incidence of cyberbullying. Also, to diminish the incidence of cyberbullying, teachers need to understand the reasons for such behavior, strengthen guidance offered to students, debunk myths pertinent to the Internet and cyberbullying, emphasize the harm done to victims of cyberbullying, and educate students about the consequences of cyberbullying from a legal standpoint.

Additionally, focusing on students of different Internet addiction levels, this study examined the influence of cyberbullying attitudes on the behaviors. The results indicate that students who are addicted to the Internet showed the influence of higher cyberbullying attitudes on the behaviors and explanatory power. Social media (e.g., Facebook, Plurk) and online games are the most participated online activities for Internet addicts. These activities are also where teenager cyberbullying happens. When students are playing online games or chatting via social media, they are exposed in the cyberbullying environment. Cyberbullying incidents are no rare cases for students to witness. Therefore, it is suggested that teachers should develop correct attitude toward Internet usage, which parents should also pay attention to. When a problem occurs, it is recommended to reach related organizations or schools for help to decrease the impacts led by different Internet addiction levels.

### Limitations and Future Research

There are several limitations in this study that should be addressed in future studies. First, this study focuses on the status quo of attitude toward cyberbullying and cyberbullying behavior, explores associations of attitude toward behavior on cyberbullying behavior in gender, different internet access times, and Internet addiction situations. *The results showed gender, different internet access times, and levels of Internet addiction, the influence of cyberbullying attitudes on the behaviors. In the research, it was not considered whether the different internet access times, and levels of Internet addiction as variables precede (or predict) the cyberbullying behaviors.* Future research should treat Internet access time and Internet addiction as antecedent variables in order to fully examine their effects of cyberbullying behaviors. Second, this study did not specifically classify the objects into groups of Internet addiction and non-Internet addiction. Therefore, we suggest using experimental designs to examine the differences between Internet addictions and non-internet addictions, so as to provide references to senior and vocational high school teachers, parents, and school managers Third, while we used cross-sectional data in this study, for it is useful for correlation purposes, causal relations cannot be concluded from such data. Fourth, previous studies’ participants have reported cyberbullying behavior according to their own personal experience. Participants are more likely to evade such questions so as to avoid judgment, causing CMV. This study uses the self-report inventory, but the items in the questions are from a third person point of view, measuring cyberbullying behavior the participant saw or heard of online. Finally, one potential limitation of this study is that we measured variables in only one timeframe using self-report questionnaires, suggesting the potential for CMV. We tested for CMV using the Harman one factor test, which showed that all constructs explained roughly an equal amount of variance, suggesting that common method bias was not an issue in this study. This study also undertakes a large data collection and sample to circumvent the problem of CMV.

## Ethics Statement

The study procedures were carried out in accordance with the APA Ethics Code. All procedures performed in studies involving human participants were in accordance with the ethical standards of the institutional and with the 1964 Helsinki declaration and its later amendments or comparable ethical standards. All subjects were informed about the research, and all provided informed consent. Given the subject matter, no ethical external approval was required under Taiwan law. All participants in the study were received the opportunity to refuse participation at any time without consequences.

## Author Contributions

C-MC: data collection, concept and design, interpretation of data, writing up; T-KY: obtaining funding, statistical analysis, interpretation of data, study supervision. All authors wrote the manuscript together and approved the final manuscript.

## Conflict of Interest Statement

The authors declare that the research was conducted in the absence of any commercial or financial relationships that could be construed as a potential conflict of interest.
